# Effect of Proton-Pump Inhibitor on Survival in Chinese Patients with De Novo Metastatic Hormone-Sensitive Prostate Cancer Receiving Upfront Combinatorial Docetaxel Treatment

**DOI:** 10.3390/cancers17172879

**Published:** 2025-09-01

**Authors:** Chris Ho-Ming Wong, Jamie Tsun Chiu, Ivan Ching-Ho Ko, David Ka-Wai Leung, Brian Siu, Cheuk-Kin Kevin Cheng, Yung-Yung Joycelyn Lim, Hiu Tung Mok, Chun-Fai Brian Kwok, Cheuk Yi Tang, Steven Chi-Ho Leung, Kang Liu, Peter Ka-Fung Chiu, Jeremy Yuen-Chun Teoh, Chi Fai Ng

**Affiliations:** 1 SH Ho Urology Centre, Department of Surgery, The Chinese University of Hong Kong, Hong Kong SAR, China; chriswong@surgery.cuhk.edu.hk (C.H.-M.W.);; 2 Department of Urology, Medical University of Vienna, 1090 Vienna, Austria; 3 Li Ka Shing Institute of Health Sciences, The Chinese University of Hong Kong, Hong Kong SAR, China

**Keywords:** proton-pump inhibitor (PPI), prostate cancer, metastatic hormone-sensitive prostate cancer (mHSPC), docetaxel, androgen deprivation therapy (ADT), drug–drug interaction

## Abstract

**Objective:** There is a growing indication of the effect of proton-pump inhibitors (PPI). Drug–drug interaction between PPI and anti-tumoral treatment has been postulated. Existing analyses from trial data have shown that PPI usage was associated with inferior survival in metastatic hormone-sensitive (mHSPC) and castration-resistant prostate cancer (mCRPC) patients treated with abiraterone and androgen deprivation therapy (ADT). The interaction between PPI and chemotherapy remains, however, unexplored. We seek to investigate the impact of PPI usage in mHSPC patients treated with upfront docetaxel and ADT. **Patients and Methods:** A prospectively maintained database was retrieved. Patients diagnosed with de novo mHSPC from 2016 to 2022 treated with upfront docetaxel (defined as being delivered within 6 months of ADT initiation) were included. The cohort was classified into those on PPI versus those who were not. A landmark analysis was performed at 6 months from treatment initiation. Outcomes included overall survival (OS), CRPC-free survival, and PSA kinetics. Multivariate Cox regression analysis was performed on survival outcomes. **Results:** One hundred and one patients were included in the study, with 79 patients in the No-PPI group and 22 in the PPI group. The median follow-up duration was 39.3 and 29.6 months, respectively. The proportion of patients that reached a PSA nadir of <0.1 ng/dL was higher in the non-PPI group (19.0% vs. 4.5%, *p* = 0.041). PPI usage was associated with inferior OS (OR = 2.28; 95%CI = 1.112–4.675; *p* = 0.025). In multivariate regression analysis, PPI usage was the only statistically significant factor for OS (*p* = 0.036). **Conclusions:** PPI usage was associated with inferior oncological outcomes in de novo mHSPC patients treated with upfront docetaxel. Its judicious use is advised.

## Simple Summary

This study looked at men with newly diagnosed metastatic prostate cancer treated with standard therapy: androgen deprivation plus early systemic therapy. It asked whether taking proton-pump inhibitors (PPIs)—common heartburn medications—affected cancer outcomes. Compared with non-users, PPI users were less likely to have a very low PSA —which indicated inferior cancer control—after treatment and had worse overall survival. After adjusting for other factors, PPI use remained the only variable linked to poorer survival. While the study is observational and cannot prove cause, it adds to growing evidence that PPIs may interfere with prostate cancer treatments. For patients on systemic therapy and hormonal therapy, PPIs should be used only when truly necessary, and alternatives or deprescribing should be considered in consultation with clinicians.

## 1. Introduction

Prostate cancer is the second most commonly found malignancy for men worldwide, with its prevalence increasing with age [1]. Over the years, considerable efforts have been made to investigate the preventive effects of medications such as 5-alpha reductase inhibitors and statins in reducing the likelihood of prostate cancer [2,3]. Another commonly prescribed class of drugs in the general population is proton-pump inhibitors (PPIs). The long-term use of PPIs has increased due to their growing list of indications [4].

In the past decade, increasing interest has been shown in understanding the potential risks associated with prolonged PPI use [5]. Population-based analyses and systematic reviews have suggested an association between the use of PPI use and the incidences of solid-organ malignancies, particularly gastrointestinal cancers such as gastric and oesophageal cancer [6]. In advanced prostate cancer patients, the majority suffer from multiple comorbidities and are often on polypharmacy regimens, frequently necessitating PPI use [7]. Existing studies have demonstrated a deleterious interaction between PPIs and abiraterone when used in juncture with androgen deprivation therapy (ADT) in both metastatic hormone-sensitive prostate cancer (mHSPC) and metastatic castration-resistant prostate cancer (mCRPC) settings [8,9,10]. However, docetaxel (DOC), another widely used chemotherapeutic agent in combination with ADT, has not been adequately studied regarding its interaction with PPIs [11]. Therefore, this study aims to investigate the impact of PPI use on the efficacy of ADT intensified with DOC in patients with mHSPC.

## 2. Materials and Methods

### 2.1. Data Acquisition

We retrieved data from the Hong Kong Prostate Cancer Study Group Database, a prospective registry that includes consecutive prostate cancer diagnosed from five hospitals in Hong Kong. The database was registered on ClinicalTrials.gov (NCT03344835). For this study, we identified consecutive patients diagnosed with de novo mHSPC between 2016 and 2022. Inclusion criteria were limited to patients treated with upfront intensification using DOC. Upfront usage was defined as DOC being initiated within 6 months from ADT. We excluded cases with missing data during follow up.

### 2.2. Cohort Information

Baseline and follow-up data were analyzed for this study. Baseline characteristics were age, prostate-specific antigen (PSA) levels prior to treatment initiation, biopsy specimen Gleason scores from biopsy specimens, the choice of upfront intensification agent, the presence of high-volume or high-risk disease, and the ECOG performance status of the cohort. ADT use, including luteinising-releasing hormone (LHRH) agonist, LHAH antagonist, and bilateral surgical orchidectomy, was included. For upfront DOC, six cycles of treatment given at 3-weekly intervals at a dose of 75 mg/m^2^ was the standard regimen.

The LATITUDE trial criteria [12] were used for the definition of high-risk disease, which included two out of three conditions: three or more sites of bone metastasis, any visceral metastasis, or ISUP grade 4 pathology or higher (8). CHAARTED trial criteria were followed for the definition of high volume disease [13], including the presence of visceral metastases or four or more bone lesions, with at least one lesion located outside the vertebral bodies and pelvis (7). Follow-up data collected include subsequent PSA levels, biochemical progression to CRPC, and mortality outcomes.

### 2.3. Classification of Cohort and Outcomes of Study

The cohort was stratified based on PPI use. In Hong Kong, the PPIs prescribed included pantoprazole, lansoprazole, esomeprazole, and omeprazole. Patients who had received either one of the PPIs at the initiation of androgen deprivation therapy (ADT) were classified into the PPI group. In contrast, the remaining patients were categorized into the No-PPI group. A landmark analysis at 6 months was performed for the outcomes, including cases that had not reached the respective endpoints. The primary outcomes of the study were the oncological outcomes: overall survival (OS), castration-resistant prostate cancer (CRPC)-free survival, and cancer-specific survival (CSS). The secondary outcomes were PSA kinetics, including time to PSA nadir (defined as the lowest PSA value recorded following the initiation of treatment), the proportion of patients achieving a PSA nadir of <0.1 ng/mL, the proportion achieving a PSA nadir of <0.2 ng/mL, the proportion achieving a PSA reduction of ≥95% from the pre-therapy PSA level (PSA95), and the proportion achieving a PSA reduction of ≥90% (PSA90) [14]. CRPC was defined according to the Prostate Cancer Working Group 3 (PCWG3) criteria [15].

### 2.4. Statistical Analysis

Methodology in statistical analysis followed existing recommendations [16]. Categorical variables were presented as counts and percentages, while continuous variables were reported as medians with interquartile ranges (IQR) or mean with standard deviations (SD). Comparisons of categorical variables were conducted using the Chi-square test or Fisher’s exact test, as appropriate. For continuous variables, Student’s *t*-test or Mann–Whitney U test was applied. A two-tailed *p*-value of <0.05 was considered statistically significant. Survival outcomes were analyzed using Kaplan–Meier methodology, with group comparisons performed using the log-rank test. Multivariable Cox regression analysis was conducted to evaluate the primary outcomes and identify potential confounding factors. Variables known to influence disease outcomes were incorporated for the development of the regression model. This included PSA level prior to treatment, age, and the intensification agent offered. The number of covariates was carefully selected to prevent model overfitting in the multivariable regression analyses [17]. SPSS software (IBM) (version 29) and R (version 4.5.0) were used.

## 3. Results

From 2016 to 2022, 101 patients met the inclusion criteria and were analyzed. Of these, 79 patients (78.2%) were in the No-PPI group, and 22 patients (21.8%) were in the PPI group. The median follow-up duration was 39.3 months for the No-PPI group and 29.6 months for the PPI group. Both groups shared similar pre-therapy characteristics. All included cases were classified as high-risk and high-volume diseases. Most cases in both groups were ISUP grade 5 (70.9% vs. 77.3%, *p* = 0.893). Similarly, the proportion of patients with a Gleason score ≥ 8 on biopsy was comparable between the groups (89.9% vs. 77.3%, *p* = 0.121). A similar proportion of patients in the two group had an ECOG status ≥ 2 upon initiation of treatment (Table 1).

Table 2 summarizes the PSA-related outcomes for both groups. A significantly higher proportion of patients in the No-PPI group achieved a PSA nadir of <0.1 ng/mL compared to the PPI group (19.0% vs. 4.5%, *p* = 0.041). While numerically, more patients in the No-PPI group achieved a PSA nadir of <0.2 ng/mL, this result did not reach statistical significance (25.3% vs. 9.1%, *p* = 0.076). The two groups had no significant difference in the proportion of patients achieving PSA90 or PSA95. Out of the cohort, 82.3% of the non-PPI group (65 patients) and 81.8% of the PPI cohort (18 patients) were documented to have progressed to mCRPC. The median time to CRPC were similar (14.2 vs. 12.7 mos, *p* = 0.38).

**Figure 1:**
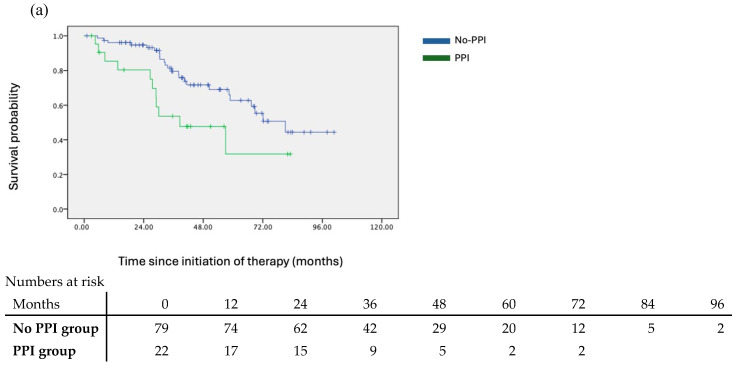

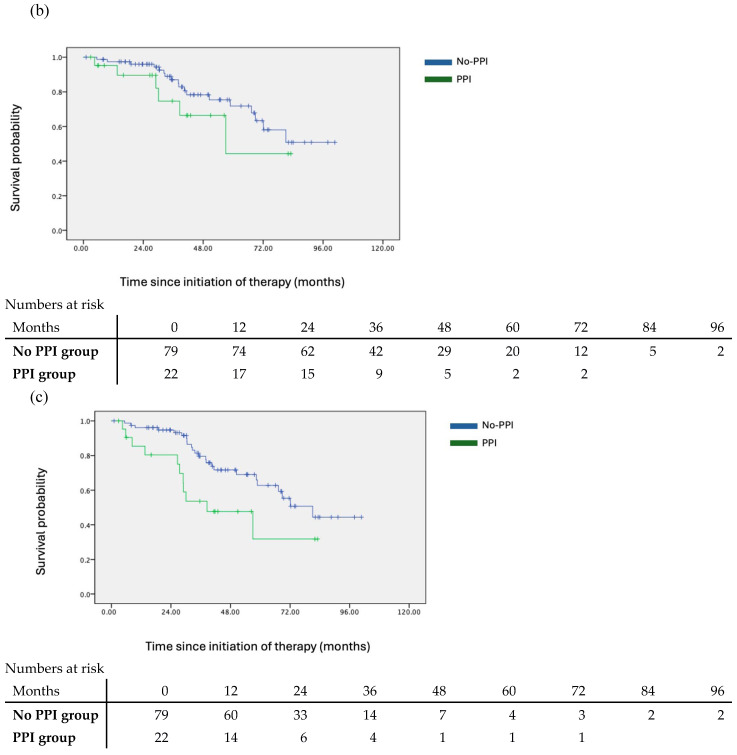
Kaplan–Meier survival curves on oncological outcomes according to PPI usage. (**a**) Overall survival, (**b**) cancer-specific survival, and (**c**) castration-resistant prostate cancer (CRPC)-free survival were plotted against time according to a dichotomous segregation of PPI usage. (**a**–**c**) There were event-free benefits observed in the non-PPI group compared to the PPI group in all of the three major outcomes described.

The No-PPI group were observed to have better OS. Kaplan–Meier survival analyses demonstrated that the median OS for the No-PPI group was 81.1 months, compared to 38.6 months for the PPI group (HR = 2.28; *p* = 0.025). While there was a trend toward better CSS and CRPC-free survival in the No-PPI group, the differences were not statistically significant. The median CSS for the No-PPI and PPI groups was not reached (NR) versus 57.0 months, respectively (HR = 1.695; *p* = 0.124), and the median CRPC-free survival was 25.2 months versus 17.0 months (HR = 1.487; *p* = 0.167) (Figure 1). Overall, 10 patients in the PPI group succumbed due to prostate cancer and the related complications, and 5 due to other causes. In the no-PPI group, 29 succumbed due to prostate cancer, and 10 from other causes. Multivariate regression analysis identified PPI usage as the only statistically significant factor associated with OS (*p* = 0.036) (Table 3).

## 4. Discussion

There has been a rising trend in the use of PPIs worldwide. With the increased prescription of PPIs in the elderly population, growing concerns have emerged regarding potential drug–drug interactions between PPIs and anti-tumour treatments [18]. PPIs are widely used to manage gastrointestinal adverse effects caused by anti-cancer therapies and to reduce the risk of stress ulcers prophylactically [5,18]. In terms of its impact on the long-term treatment of prostate cancer, previous studies have shown that PPI use can diminish the efficacy of combinatorial abiraterone in both mHSPC and mCRPC settings [8,11]. While also used as a combinatorial chemotherapeutic agent, evidence regarding the interaction between PPIs and DOC has been limited. The current analysis was one of the first, and we demonstrated that PPI usage was associated with poorer oncological outcomes in mHSPC patients treated with ADT and upfront combinatorial DOC. Notably, a hazard ratio of 2.28 was observed for overall survival in patients taking PPIs. Overall, aside from a numerically significant difference in the OS between PPI users and non-users, from the Kaplan–Meier analysis, it was also noted that the segregation of the curves began early in the first year of treatment initiation. This could in fact hint that the significance of PPI use could be valid from early on, prior to development of CRPC stage which was typically observed after up to 24 to 36 months of intensified primary treatment. Multiple mechanisms could potentially explain the correlation, and the key postulations shall be highlighted.

The first core principle could be related to the drug–drug interaction involved by PPI. Multiple mechanisms have been proposed to explain the drug–drug interaction between PPIs and chemotherapeutic agents. In a population-based analysis of 20,000 prostate cancer patients, Goldberg et al. reported a potential negative association between PPI use and prostate cancer outcomes. Over a mean follow-up duration of 8.06 years, any use of PPIs was associated with a 39.0% increased risk of prostate cancer-specific mortality and a 3.0% increase in ADT usage. PPIs have been postulated to reduce the efficacy of upfront DOC treatment by blunting its inhibitory effects on androgen-sensitive human prostate cancer cells [19,20], with elevated chromogranin A levels in CRPC patients [19] and a promotion of cell proliferation and survival in castration-sensitive prostate cancer cell lines [8]. At the molecular level, PPIs exert their effects by inducing cell-cycle progression, increasing the expression of oncoproteins (e.g., c-Myc) and anti-apoptotic proteins (e.g., Bcl-2) and inhibiting prostate phosphatases [19]. These changes could conceivably make cancer cells less susceptible to chemotherapy-induced death. Direct carcinogenic effects of PPI use have also been demonstrated in various ex vivo animal models [21,22,23], with PPI being shown to be associated with promoted cell-cycle progression, increased ErbB2 activity and PSA secretion in mice [7,24].

The interplay between PPIs, chemotherapeutic agents, and human microbiota has also gained increasing attention. Although PPIs are effective in treating acid-related disorders and are generally considered to have a favourable safety profile [25], there is growing concern that their use may alter the human microbiota. Prostate cancer has been associated with changes in the balance of gut microbiota. For instance, a reduction in the population of *Akkermansia muciniphila* (*A. muciniphila*) in the gut has been observed in prostate cancer patients. Interestingly, *A. muciniphila* levels are also reduced with PPI use, though this effect can be reversed by ADT [9]. These findings highlight the potential role of PPI-induced gut microbiota disruption in reducing the efficacy of chemotherapeutic agents. Additionally, prostate cancer at different stages has been shown to increase pro-inflammatory microbiota in the gut [26,27]. Notably, up to 20% of the gut microbiota composition differs between PPI users and non-users [28]. This further supports the hypothesis that PPI use negatively impacts treatment outcomes in mHSPC by altering the gut microbiota environment.

A potential limitation of the current study is its retrospective nature. Despite efforts to mitigate confounding factors through multivariate regression analysis, uncontrollable confounders remain inevitable. This is particularly relevant in the context of advanced malignancy, where patients often have multiple comorbidities and are subject to polypharmacy, which could influence outcomes, and specifically leads to mortality (that could confound to the oncological outcomes of OS and CSS). While our cohort was based on a real-world clinical setting, compared to well-designed trials with careful patient selection trials like the CHAARTED trial, there were more patients with relatively poor performance status in our cohort. This disproportionately high number of patients with poor performance status may affect the oncological endpoints to a different extent than well-known RCTs. Also, the next line therapies after castration resistance were not fully detailed and accounted for in this cohort, thereby potentially confounding the oncological outcomes. Methodologically, a causal relationship cannot be established, and further high-quality clinical data are required to elucidate better the effect of PPI use on the efficacy of prostate cancer chemotherapeutic agents. To be specific, these hypotheses regarding the drug–drug interaction brought about by PPI remained speculative. They were not directly supported by clinical or mechanistic data relevant to anti-tumoral treatment such as docetaxel. While we aimed to create a more homogenous cohort by including only de novo mHSPC patients treated with combinatorial DOC, we acknowledge that the relatively small sample size may limit the generalizability of our findings. The limited patient sample size, especially in the PPI subgroup, might affect the robustness of the outcomes. Additionally, PPI use was classified dichotomously in our study. We could not account for the potential impact of variables such as duration of PPI use, route of administration (oral versus intravenous), or specific types of PPI regimens. Furthermore, while PPI use can be indicated for various clinical reasons, we could not accurately determine the exact clinical indications for PPI use in individual patients within our cohort.

## 5. Conclusions

From real-world data of prostate cancer patients, the use of PPIs was found to be associated with inferior overall survival in de novo mHSPC treated with upfront docetaxel. However, further clinical studies are required to establish a causal relationship. Clinicians are advised to carefully evaluate the indication for PPI use when managing patients with mHSPC.

## Figures and Tables

**Table 1:** Baseline characteristics of the cohort grouped according to PPI usage.

	No PPI Group		PPI Group		*p* Value
	N		N		
Number of patients, *%*	79	78.2%	22	21.8%	
Follow-up duration (mos), IQR	39.3	23.6	29.6	23.1	N/A
Median age at treatment initiation (years), IQR	66	9	68	7	0.33
Mean PSA prior treatment initiation, SD	684.5	127.2	493	170.7	0.32
Gleason score ≥ 8, *%*	71	89.9%	17	77.3%	0.12
ISUP grading in prostate biopsy, %					0.90
1	1	1.3%	0	0.0%	
2	2	2.5%	1	4.5%	
3	4	5.1%	2	9.1%	
4	16	20.3%	2	9.1%	
5	56	70.9%	17	77.3%	
High-volume disease, *%*	79	100%	22	100%	N/A
High-risk disease, *%*	79	100%	22	100%	N/A
ECOG performance status ≥ 2, %	33	41.8%	10	45.2%	0.29

PPI = proton-pump inhibitor; PSA = prostate-specific antigen; ISUP = International Society of Urological Pathology; SD = standard deviation; IQR = interquartile range; mos = months; N/A = not applicable.

**Table 2:** Disease outcomes (related to PSA kinetics and progression to CRPC) of the cohort grouped according to PPI usage.

	No PPI Group		PPI Group		*p* Value
	N		N		
Median time to PSA nadir (mos), 95%CI	9.4	8.2–10.6	6.8	3.6–10	0.40
Portion of patients reaching nadir < 0.1, %	15	19.0%	1	4.5%	0.041
Portion of patients reaching nadir < 0.2, %	20	25.3%	2	9.1%	0.076
Portion of patients reaching PSA95, %	36	45.6%	9	40.9%	0.49
Portion of patients reaching PSA90, %	52	65.8%	14	63.6%	0.62
Median time to CRPC (mos), 95%CI	14.2	11.0–17.4	12.7	7.6–17.8	0.38

PSA = prostate-specific antigen; CRPC = castration-resistant prostate cancer; mos = months.

**Table 3:** Univariate and multivariate Cox regression analysis of factors associated with survival outcomes of OS, CSS and CRPC-free survival in the cohort.

	Univariate Analysis				Multivariate Analysis			
	OR	95% CI		*p* Value	OR	95% CI		*p* Value
Overall survival								
PPI usage	2.28	1.112	4.675	0.025	2.268	1.055	4.874	0.036
Age	0.973	0.923	1.026	0.32	0.992	0.933	1.054	0.79
logPSA prior treatment initiation	1.059	0.635	1.768	0.83	1.08	0.683	1.707	0.74
Gleason score ≥ 8	1.654	0.505	5.412	0.41	2.797	0.65	12.043	0.17
Cancer-specific survival								
PPI usage	1.695	0.67	4.29	0.27	1.981	0.761	5.159	0.16
Age	0.984	0.922	1.049	0.62	1.017	0.944	1.095	0.66
logPSA prior treatment initiation	1.549	0.882	2.72	0.13	1.515	0.859	2.671	0.15
Gleason score ≥ 8	3.716	0.501	27.582	0.20	3.873	0.51	29.395	0.19
CRPC-free survival								
PPI usage	1.487	0.846	2.615	0.17	1.33	0.733	2.412	0.35
Age	0.991	0.949	1.035	0.70	1.015	0.966	1.067	0.55
logPSA prior treatment initiation	1.149	0.847	1.557	0.37	1.128	0.824	1.544	0.45
Gleason score ≥ 8	1.372	0.626	3.007	0.43	1.716	0.708	4.16	0.23

PPI = proton-pump inhibitor; PSA = prostate-specific antigen, CRPC = castration-resistant prostate cancer.

## Data Availability

The data involved in the study could be made available from the corresponding author upon request.
